# Chitosan-DNA nanoparticles enhanced the immunogenicity of multivalent DNA vaccination on mice against *Trueperella pyogenes* infection

**DOI:** 10.1186/s12951-018-0337-2

**Published:** 2018-01-29

**Authors:** Ting Huang, Xuhao Song, Jie Jing, Kelei Zhao, Yongmei Shen, Xiuyue Zhang, Bisong Yue

**Affiliations:** 1 Key Laboratory of Bio-resources and Eco-environment (Ministry of Education), College of Life Sciences, Sichuan University, Chengdu, China; 2 Sichuan Key Laboratory of Conservation Biology on Endangered Wildlife, College of Life Sciences, Sichuan University, Chengdu, 610064 Sichuan China; 3 Sichuan Engineering Technology Research Center of Medical Animal, Chengdu, China

**Keywords:** *Trueperella pyogenes*, DNA vaccine, Chitosan nanoparticles, CpG motifs, Virulence factors, Multi-valency

## Abstract

**Background:**

*Trueperella pyogenes* is a commensal and opportunistic pathogen that normally causes mastitis, liver abscesses and pneumonia of economically important livestock. To develop efficacious and potent vaccine against *T. pyogenes*, chimeric gene DNA vaccines were constructed and encapsulated in chitosan nanoparticles (pPCFN-CpG-CS-NPs).

**Results:**

The pPCFN-CpG-CS-NPs consists of the *plo*, *cbpA*, *fimA*, and *nanH* gene of *T. pyogenes* and CpG ODN1826. It was produced with good morphology, high stability, a mean diameter of 93.58 nm, and a zeta potential of + 5.27 mV. Additionally, chitosan encapsulation was confirmed to protect the DNA plasmid from DNase I digestion. The immunofluorescence assay indicated that the four-chimeric gene could synchronously express in HEK293T cells and maintain good bioactivity. Compared to the mice immunized with the control plasmid, in vivo immunization showed that mice immunized with the pPCFN-CpG-CS-NPs had better immune responses, and release of the plasmid DNA was prolonged. Importantly, immunization with pPCFN-CpG-CS-NPs could significantly protect mice from highly virulent *T. pyogenes* TP7 infection.

**Conclusions:**

This study indicates that chitosan-DNA nanoparticles are potent immunization candidates against *T. pyogenes* infection and provides strategies for the further development of novel vaccines encapsulated in chitosan nanoparticles.

**Electronic supplementary material:**

The online version of this article (10.1186/s12951-018-0337-2) contains supplementary material, which is available to authorized users.

## Background

*Trueperella pyogenes* is an opportunistic pathogen causing mastitis, abscesses, and pneumonia, and can be isolated from the mucous membranes of domestic ruminants and wild animals [1–4]. Previous studies have shown that *T. pyogenes* expresses several known and putative virulence factors including haemolytic exotoxin pyolysin (PLO), collagen-binding protein (CbpA), neuraminidases, and fimbriae that play important roles during infection [4–6]. PLO is a primary virulence factor of *T. pyogenes* and capable of lysing immune cells [7]. CbpA is expressed on the surface of *T. pyogenes* cells that can promote the adherence and subsequent colonization of *T. pyogenes* to collagen-rich tissue [5]. The two neuraminidases (NanH and NanP) and several fimbriae were found to play an indispensable role in promoting adhesion of the organism to host epithelial cells [2, 8, 9].

Although antibiotic therapy is available for the treatment of *T. pyogenes* infection, drug resistant isolates pose a major challenge to veterinary practice and a potential threat to human health due to the mobile genetic elements and antibiotic selective pressure [10–12]. The development of an effective vaccine against *T. pyogenes* would therefore facilitate the prevention and treatment of such infections. Genetic immunization is a promising strategy to induce protective immune responses against a great variety of viral, bacterial, and parasitic pathogens infections [13–16]. Compared with the conventional live or subunit vaccines, DNA vaccines have several potential advantages such as being easier to manufacture, having greater stability, and conferring potential safety [17]. More importantly, a major potential advantage of DNA vaccines is that it may be possible to mix several encoding antigens from different strains of a single pathogen or from multiple pathogens. Moreover, it can induce immune responses against multivalent antigens and effectively protect the host against a variety of infections. However, a number of clinical trials have shown that the magnitude of immune responses elicited by DNA vaccines are generally weak [18], especially in large animals. This may be due to the amount of DNA required for effective immunization being much greater. Thus, the host immune response to DNA vaccine needs to be enhanced. CpG dinucleotides are selectively methylated in vertebrate DNA, but are present at the expected frequency (1/16 bases) and unmethylated in bacterial DNA [19, 20]. CpG DNA, as a molecular adjuvant, can induce T helper 1 (Th1)-like cytokine responses by stimulating antigen-presenting cells via toll-like receptors [21–23]. A previous study also suggested that CpG DNA could markedly enhance the systemic immune responses against inactivated H9N2 avian influenza viruses when administered to ducks [24].

Except for CpG DNA, the nanoparticles prepared by biomaterials can also offer several advantages to improve the efficacy of DNA vaccine. For instance, they can protect antigens from degradation in vitro and in vivo, limit systemic distribution, and thereby reduce the dose and probable side effects [25]. Chitosan is a natural biodegradable polysaccharide extracted from crustacean shells and nontoxic in both experimental animals and humans [26–28]. Previous studies have shown that chitosan is a promising DNA vector with sustained-releasing ability, and it can greatly enhance transfection and expression efficiency of DNA vaccines, thereby increasing their bioavailability [29–31]. Interestingly, chitosan can also promote dendritic cell maturation by inducing type I interferons (IFNs) and enhance antigen-specific Th1 responses in a type I IFN receptor-dependent manner [32].

In order to enhance the efficacy of a DNA vaccine against *T. pyogenes* infections, we constructed a chimeric gene DNA vaccine encapsulated in chitosan nanoparticles (pPCFN-CpG-CS-NPs) by a complex coacervation method. Stability and in vitro expression of the chitosan nanoparticles were studied by DNase I digestion and transfection. The immune responses elicited in specific pathogen-free (SPF) mice by the pPCFN-CpG-CS-NPs were evaluated. Furthermore, protective potential of pPCFN-CpG-CS-NPs was assessed by challenging with *T. pyogenes*. Finally, we found that pPCFN-CpG-CS-NPs induced significantly higher host immune responses and had better protective effects against the challenge of *T. pyogenes* infections based on a mouse model.

## Methods

### Bacterial strains, cells and growth conditions

The moderately virulent *T. pyogenes* TP8 and highly virulent *T. pyogenes* TP7 strain were isolated from the abscess of *Moschus berezovskii* (forest musk deer) [4]. *M. berezovskii* has been farmed in China since the 1950s, and abscess-related diseases are key factors preventing sustainable and increasing captive-commercial populations. The two strains were cultivated on a blood agar medium at 37 °C with 5% CO_2_ for 48 h. Human embryonic kidney cells (HEK293T) and RAW264.7 murine macrophages were kindly provided by Dr. Rui Peng (Sichuan University) and were grown in Dulbecco’s Modified Essential Medium (DMEM) containing 10% fetal bovine serum (FBS) at 37 °C with 5% CO_2_.

### Multi-epitope genes selection

The nucleic acid sequences of *plo*, *cbp*A, *fimA*, and *nanH* gene of the *T. pyogenes* TP8 strain were obtained from GenBank (CP007003). The epitope sequences of the four genes were analyzed according to IEBD Analysis Resource (http://tools.immuneepitope.org/bcell/) [33] or BepiPred 1.0 Server (http://www.cbs.dtu.dk/services/BepiPred/) [34] and reported references [5, 8, 35, 36], and then thirteen B cell epitopes from PLO, CbpA, FimA, and NanH proteins were selected (Table 1).

**Table 1:** Character of each selected epitope in the design

Protein	Epitope	Start-end	Peptide sequence	Length
PLO	1	52–74	KVDLKSAQETNETSVDKYIRGLK	23
	2	122–166	AFDANNAHVYPGALVLANKDLAKGSPTSIGIARAPQTVSVDLPGL	45
	3	487–505	VEAGEATGLAWDPWWTVIN	19
CbpA	4	150–346	DRGTTRTLKVGNTIVKIAHGNGGDRGVFAWKTGIMYGDFKPGYVTWSLRANINGDVWPGGPVKIVDKLGEGQILDGSGISIALYWHGQQHQTHKLTWSSIDDFLNDPYYGRNKGTSIAYNKDDGTINIDIPHEVISEKEFSFTYDAKITDETLEEFKNHATFDFYENQIKKQITDTFTVRNPKASGGIEGKTTASVN	197
FimA	5	158–196	MPKGDNEWVYDVHAYPKNKLTEPGVPTKTASEPTKFVPG	39
	6	384–414	YVGKNESDSKDYCLKETAAPAGYVLDPVGRT	31
	7	432–446	VKVEGPDLPLTGAQG	15
NanH	8	319–321	RIP	3
	9	358–369	RRSKDGGKTWGP	12
	10	439–448	SSSKDNGYTW	10
	11	507–534	SDDHGKTWQSGQFASANAGAPAGQRWNF	28
	12	565–574	ATSSDGGVNW	10
	13	626–636	KPNNRVDGKVK	11

### Design and construction of the multi-epitope chimeric DNA vaccine

The 13 multi-epitope minigenes were paralleled as a single chimeric gene separated from each other with the GGGGS/(Gly)_6_ linker [37] and were synthesized by BGI company (Shenzhen, China). The constructs included a Kozak sequence at the N-terminus and CpG ODN1826 motif at the end of the chimeric gene to enhance immune response [38]. The multi-epitope chimeric gene was then incorporated into expression vector pVAX1 (Invitrogen, USA) designated as pPCFN-CpG, which was confirmed by endonuclease digestion assay and DNA sequencing. To investigate the immune response to different multi-epitope chimeric DNA vaccine, the pPCFN-CpG plasmids were digested with *Hin*dIII and *Eco*RV, followed by cloning into the same sites of the vector pVAX1, resulting in a recombinant plasmid pVAX1-PCFN. Likewise, the pPCFN-CpG plasmids were single digested with *Kpn*I or *Eco*RI, followed by cloning into the same sites of the vector pVAX1, resulting in the recombinant plasmid pVAX1-PCF and pVAX1-PC. All the plasmids were transformed into *Escherichia coli* DH5α and purified using Endo-free Maxiprep kit (Qiagen, Hilden, Germany) as previously described [39].

### Preparation of chitosan solutions and plasmid DNA solutions

Chitosan (with a molecular weight of 71.3 kDa and deacetylation degree of 80%) was purchased from Sigma-Aldrich (Sigma, St Louis, MO, USA). Chitosan solutions of 1.0% were prepared by slowly dissolving 1.0 g chitosan in an aqueous solution of 1.0% acetic acid and adjusted to a final concentration of 250 μg/mL with acetate (5.0 mmol/L). The plasmid DNA solutions were diluted to a final concentration of 100 μg/mL pPCFN-CpG with Na_2_SO_4_ solution (5.0 mmol/L) [40].

### Preparation of the plasmid DNA-chitosan nanoparticles

The plasmid DNA chitosan nanoparticles were prepared by a complex coacervation method as previously described by Boyoglu et al. [41]. Briefly, 500 μL chitosan solutions with an equal volume of the plasmid DNA solutions were heated in a water bath of 55 °C for 30 min. Subsequently, an equal volume of plasmid DNA solution was quickly transferred to the chitosan solutions and vortexed for 30 s at 2500 r/min. The plasmid DNA chitosan nanoparticles were collected by centrifugation at 2500 r/min for 10 min at 4 °C and the precipitate was resuspended in phosphate buffered saline (PBS, pH 7.4). These nanoparticles were simply named as the pPCFN-CpG-CS-NPs.

### Characterization of the pPCFN-CpG-CS-NPs

The pPCFN-CpG-CS-NPs were examined by Tecnai G^2^ F20 transmission electron microscopy (TEM) (FEI, Houston, TX, USA) to assess the morphological and surface characteristics as previously described [40]. The particle size and zeta potentials of the pPCFN-CpG-CS-NPs were measured using a Zeta Sizer 2000 from Malvern Instruments (Malvern, UK). Samples were diluted with pure water and the measurements were performed at a scattering angle of 90° and a temperature of 25 °C. The diameter was calculated from the autocorrelation function of the intensity of light scattered from particles, assuming a spherical form of the particles.

### Stability of the pPCFN-CpG-CS-NPs

Both 1.35 μg of naked plasmid DNA (5.0 mmol/L Na_2_SO_4_) and the pPCFN-CpG-CS-NPs suspension containing 1.35 μg of plasmid DNA were incubated with DNase I (1.0 U/mL) at 37 °C for 30 min according to a previous study [40]. Briefly, the reaction was stopped by adding 100 μL of termination solutions (400 mmol/L NaCl, 100 mmol/L ethylenediaminetetraacetic acid [EDTA], pH 8.0) at 65 °C for 10 min. Subsequently, 16 μL of chitosanase (0.2 U/mL) and 4.0 μL of lysozyme (0.2 U/mL) were added and incubated in a 37 °C water bath for 1 h. The pPCFN-CpG-CS-NPs suspension and the naked plasmid DNA were used as negative controls. The integrity of plasmid DNA was analyzed using 0.8% agarose gel electrophoresis.

### Immunofluorescence assay

The recombinant plasmids pPCFN-CpG, pVAX1-PCFN, pVAX1-PCF, and pVAX1-PC were prepared as described above. The plasmids were dissolved in 0.1 M PBS to a final concentration of 500 μg/mL. When HEK293T cells were cultured to approximately 80% confluence in 12-well plates, 1 μg of pPCFN-CpG, pVAX1-PCFN, pVAX1-PCF and pVAX1-PC or empty vector pVAX1 were transfected into the cells using lipofectamine 2000 (Invitrogen, Carlsbad, CA, USA). Additionally, the pPCFN-CpG-CS-NPs containing 1 μg of plasmid DNA were directly transfected into the cells when HEK293T cells were cultured to approximately 80% confluence. After 30 h post transfection, the expressions of PLO proteins were detected by immunofluorescence assays (IFA) as described previously [39]. Briefly, the cells fixed with 4% methanol were incubated with the primary mouse polyclonal antibody against PLO, which was prepared according to the protocol [42] for 1.5 h (1:200 dilution in 0.1 M PBS). After being washed 3 times with PBS, the cells were incubated with fluorescein isothiocyanate (FITC)-labeled goat anti-mouse IgG (Origene, Rockville, MD, USA) for 1 h in the dark (1:200 dilution in 0.1 M PBS). Cells were washed with PBS and were incubated with Hoechst 33,342 dye (1 μg/mL; Sigma) for 5 min in the dark. The green and blue fluorescence signals were observed under inverted fluorescence microscope (Leica, Germany). All experiments were performed in triplicate.

### Western blotting

To investigate the activation of CpG, we measured the expression of Toll-like receptor 9 (TLR9) and myeloid differentiation primary response 88 (Myd88) by using western blotting [22]. The pPCFN-CpG, pVAX1-PCFN, and empty vector pVAX1 were transfected into the RAW264.7 using lipofectamine 2000. After 36 h, the samples derived from RAW264.7 were collected and lysed in RIPA buffer, separated by electrophoresis on 12% SDS-PAGE gels and transferred to nitrocellulose (GE Amersham Biosciences, Piscataway, NJ, USA). Proteins were detected by western blotting using primary antibodies (Abs) (Mouse monoclonal Abs against GAPDH, Myd88, TLR9) at a concentration of 1/1000 (Santa Cruz Biotechnology, Santa Cruz, CA, USA) and were incubated at 4 °C overnight. Labeling of the first Abs was detected using goat anti-mouse Abs conjugated to HRP (Santa Cruz Biotechnology) and detected by using ECL reagents.

### Quantitative PCR

To confirm the activation of CpG, the relative mRNA levels of TLR9 and Myd88 from the RAW264.7 were detected by quantitative PCR (qPCR). Specific primers (Table 2) for TLR9, Myd88, and GAPDH were designed by using Primer-BLAST software (http://www.ncbi.nlm.nih.gov/tools/primer-blast/), based on the consensus of sequences from GenBank. The pPCFN-CpG, pVAX1-PCFN, and empty vector pVAX1 were transfected into the RAW264.7 using lipofectamine 2000. The total RNA from the RAW264.7 was extracted by using RNA isolation kit (Foregene, Chengdu, China) after 36 h, followed by reverse transcription and qPCR using a One-Step RT-PCR kit (TransGen, China) according to the manufacturer’s instructions. All experiments were performed in triplicate. Gene expression was calculated by using the 2^−Δ*CT*^ method and normalized to GAPDH levels in each sample.

**Table 2:** Primers for quantitative PCR used in this study

Gene assayed	Primer sequence
*TLR9*	5′-TGGGCCCATTGTGATGAACC-3′ (forward)
	5′-CTTGGTCTGCACCTCCAACA-3′ (reverse)
*Myd88*	5′-GCACCTGTGTCTGGTCCATTG-3′ (forward)
	5′-TCTGTTGGACACCTGGAGACA-3′ (reverse)
*GADPH*	5′-CCCACTCTTCCACCTTCGAT-3′ (forward)
	5′-CTTGCTCAGTGTCCTTGCTG-3′ (reverse)

These primers were designed in this study

### Mice models

All animal protocols were performed in accordance with the permission of the Institutional Animal Care and Use Committee guidelines (Sichuan University). We used 6- to 8-week-old, out-bred SPF female Kunming (KM) mice (Dashuo biotechnology, Chengdu, China), which were divided into seven groups randomly (11 mice per group). After anesthetizing with a ketamine/xylazine mixture, the mice were injected in the medial rectus muscle with plasmids (50 μg/mouse) pPCFN-CpG, pVAX1-PCFN, pVAX1-PCF and pVAX1-PC as well as 50 μL PBS or the pPCFN-CpG-CS-NPs and chitosan nanoparticles solutions (CS-NPs). The mice were boosted once on day 21 after primary immunization by using the same inoculation protocols.

### IgG antibody in serum

Peripheral blood was collected from the tail vein of mice before vaccination and at 21, and 42 days post immunization (dpi). The blood collected was stored at 37 °C for 1 h and centrifuged for serum collection. The levels of PLO-specific antibody were analyzed to determine the humoral immune response by indirect ELISA assay as previously described [39]. To determine whether this type of vaccine was dependent on Th1 type immune response, the serum antibody titers IgG1 and IgG2a subtype were also monitored at regular intervals by ELISA. All experiments were performed in triplicate.

### Detection of cellular immune response

To investigate the cellular immune response induced by the plasmids, the proliferation of lymphocytes, changes of CD4^+^ and CD8^+^ T lymphocyte number and the levels of IFN-γ, IL-2, and IL-4 were assessed. The lymphocytes from the spleen or peripheral blood were isolated and purified using lymphocyte separation solution (TBD, China) as previously described [39]. The proliferation of the lymphocytes was determined by the MTT (3-[4,5-dimethylthiazol-2-yl]-2,5-diphenyl tetrazolium bromide) assay according to a previous report [39]. All treatments were performed in triplicates.

The CD4^+^ and CD8^+^ T lymphocyte cells were sorted by flow cytometry as previously described [39]. Briefly, lymphocytes were incubated with FITC-conjugated anti-CD4^+^ T cell antibody and phycoerythrin (PE)-conjugated anti-CD8^+^ T cell antibody (1:1000 dilution) (Sungene, China) at 4 °C for 35 min. After incubation, the cells were washed with cold PBS 3 times, then suspended in PBS and subjected to flow cytometry.

The IFN-γ, IL-2, and IL-4 from the suspension of the spleen lymphocytes were detected using ELISA kits (ChengLinBio, China) according to the manufacturer’s instructions. The levels of the IFN-γ, IL-2, and IL-4 were analyzed according to their corresponding standard curves.

### Challenge experiments

To investigate the protective efficacy of different multi-epitope DNA vaccine, all groups were challenged with 3.7 × 10^8^ CFU *T. pyogenes* TP7 and *T. pyogenes* TP8 respectively by intraperitoneal injection 3 weeks after the second immunization. The mortality of the challenged mice was monitored for the subsequent 30 days. The bacterial burdens in the liver and peritoneal fluid (PF) were detected at day 7 post infection as previously described [39]. Briefly, mice were euthanized at day 7 post infection. PF was obtained by lavage with 3 mL of PBS. The liver was aseptically removed and was macerated by passage through a 3-mL syringe. Serial dilutions of PF and liver were incubated on brain heart infusion (BHI, BD Difco, NJ, USA) agar medium containing 5% fetal bovine serum (FBS, GE Healthcare, NJ, USA.) at 37 °C for 48 h, and bacterial viable counts were determined. Infection was measured as either mortality or the presence of ≥ 500 CFU of *T. pyogenes* per g of liver and per mL of PF during necropsy.

### Histological analysis

After procedures for the necropsy at day 7 post infection, the livers of the seven groups of immunized mice were aseptically harvested and fixed in 10% formalin. The paraffin-embedded tissue sections were prepared on a rotary microtome and stained with hematoxylin–eosin by using standard techniques [43]. All sections were examined by light microscopy. Triplicates were completed for each control and sample.

### Statistical analysis

Data and statistical tests were analyzed using GraphPad Prism 5.0. Means were compared by using a one-way analysis of variance (ANOVA), followed by a Tukey–Kramer post hoc test using a 95% confidence interval. A Chi square test with Yates’ correction was used to compare the survival rates between immunized mice and the control group. Differences were considered significant at *p* < 0.05 and very significant at *p* < 0.01.

## Results

### Construction and identification of pPCFN-CpG

**Fig. 1:**
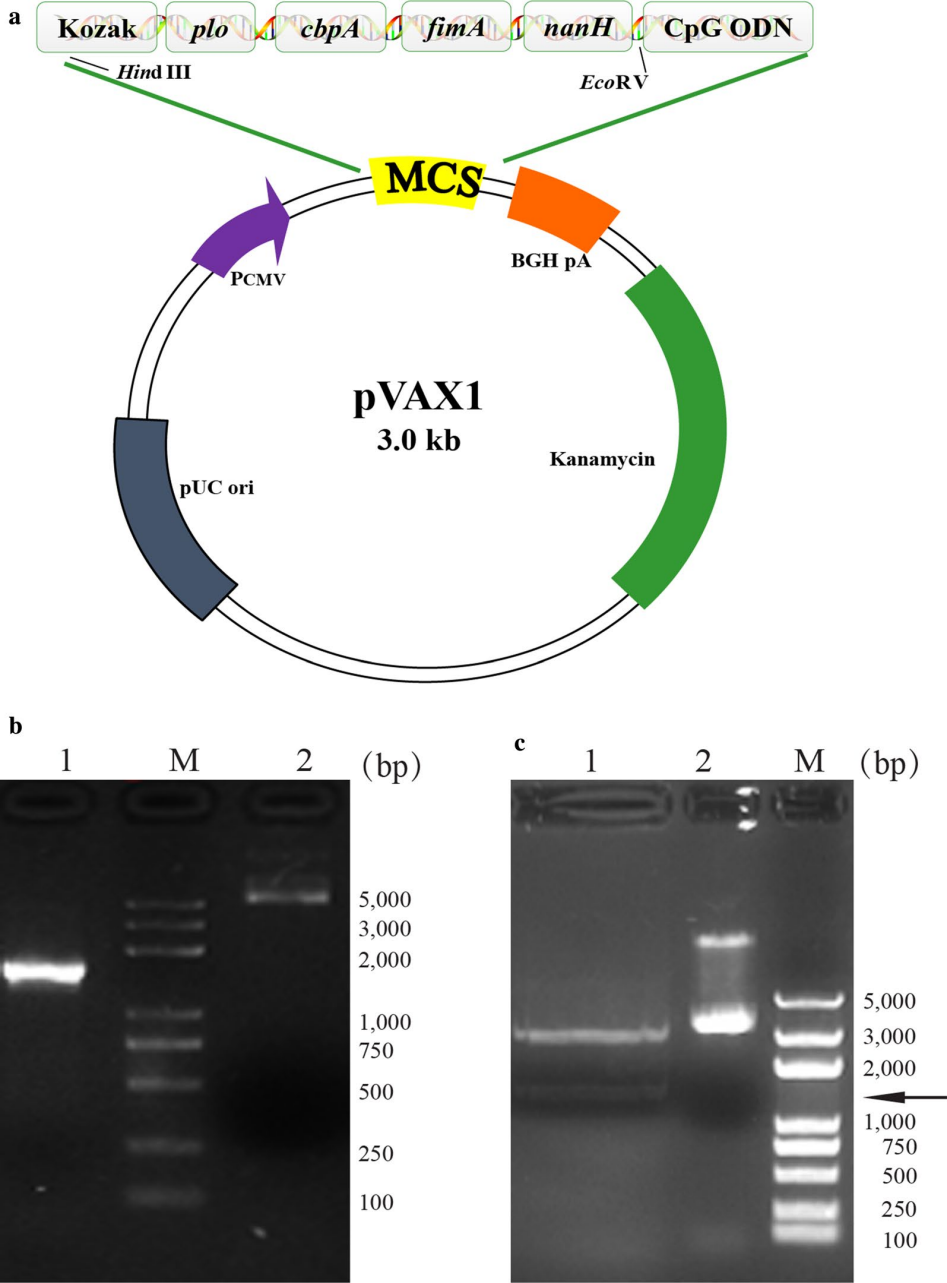
Identification of the multivalent DNA plasmid pPCFN-CpG. **a** Schematic representation of pPCFN-CpG. The chimeric gene containing four different virulence genes was inserted into pVAX1 utilizing *Hin*dIII and *Eco*RV sites. **b** The multivalent DNA plasmids were separated by electrophoresis. Lane 1, pVAX1 empty plasmid; Lane 2, pPCFN-CpG plasmid; Lane M, DNA marker-DL5000. **c** The digestion products were separated by electrophoresis. Lane 1, two fragments after restriction enzyme digestion with *Hin*dIII and *Eco*RV; Lane 2, pPCFN-CpG plasmid. Lane M, DNA marker-DL5000. The position of the 1482 bp band is indicated by the arrow. pPCFN-CpG, the DNA plasmid contains the epitope of *plo*, *cbpA*, *fimA*, and *nanH* gene of *T. pyogenes* and CpG ODN1826 motif

As shown in Fig. 1a, b, the chimeric gene containing four different virulence genes was cloned into the eukaryotic expression vector pVAX1, resulting in the DNA vaccine plasmid pPCFN-CpG. The chimeric gene sequence from the recombinant plasmid pPCFN-CpG shared 100% identity with the strain *T. pyogenes* TP8 by DNA sequencing (data not shown). The constructed plasmid was subjected to digestion with *Hin*d III and *Eco*RV. Electrophoretic separation of the digestion products about 1482 bp (Fig. 1c) demonstrated that the construction of the recombinant plasmid was successful.

### Chitosan enhanced stability of the pPCFN-CpG

**Fig. 2:**
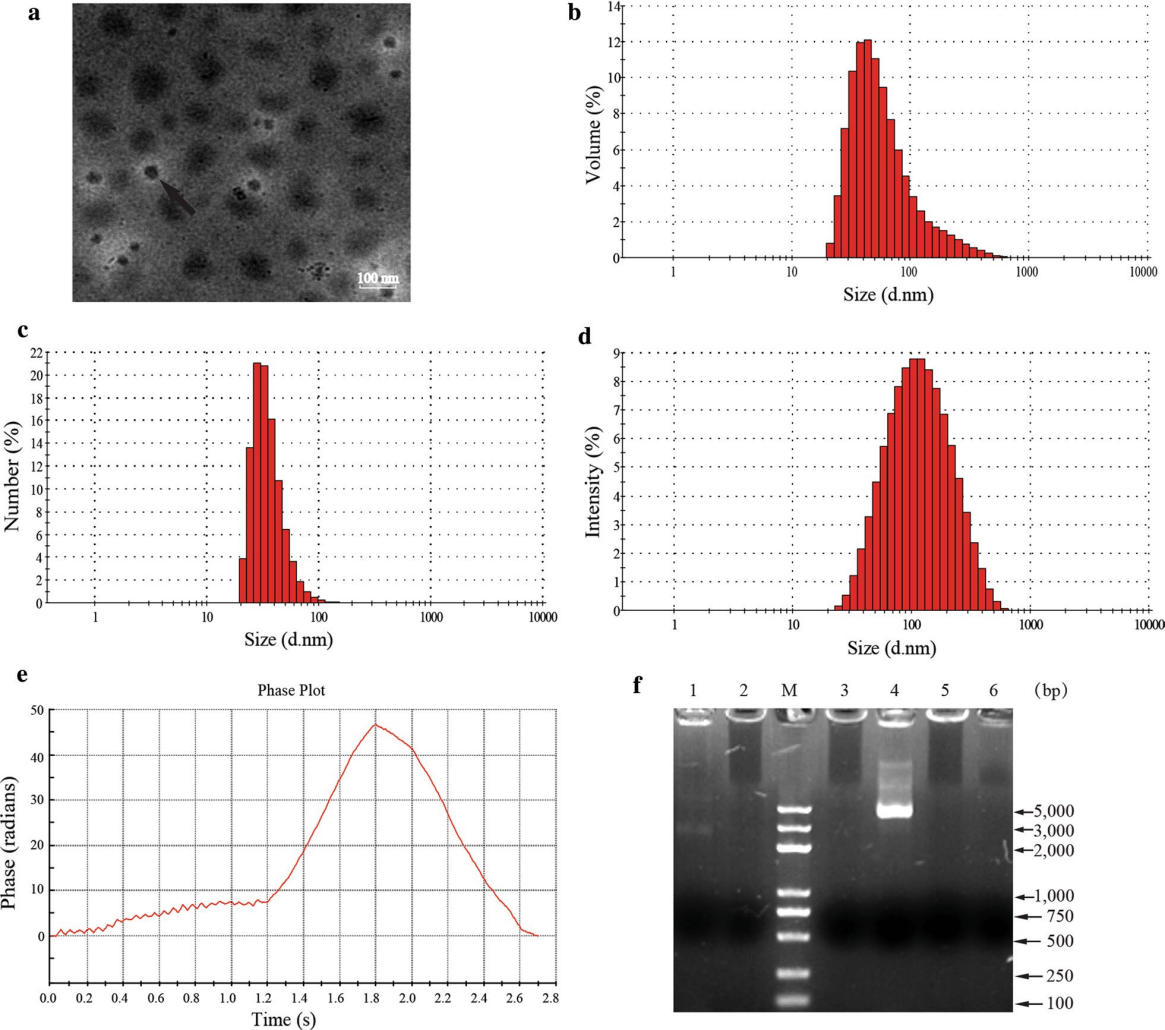
Characterization and stability of the pPCFN-CpG-CS-NPs. Transmission electron microscopy micrograph of the pPFCN-CpG-CS-NPs (magnification 30,000×). **a** pPCFN-CpG-CS-NPs at pH 5.5 is indicated by the arrow. **b**–**d** Measurement of these particles showed a narrow distribution of the pPCFN-CpG-CS-NPs, and the average diameter was 93.58 nm. **e** Measurement of these particles showed a Zeta potential of + 5.27 mV. **f** Stability analysis of the plasmid DNA after encapsulation in the chitosan nanoparticles. Lane 1: pPCFN-CpG-CS-NPs treated by DNase I and chitosanase; Lane 2: pPCFN-CpG-CS-NPs treated by DNase I; Lane 3: untreated chitosan encapsulated plasmid pPCFN-CpG; Lane4: untreated naked plasmid pPCFN-CpG; Lane 5: pPCFN-CS-NPs treated by DNase I; Lane 6: naked plasmid pVAX1-PCFN treated by DNase I; M: DNA marker DL 5000. pPCFN-CpG-CS-NPs, the pPCFN-CpG DNA plasmid encapsulated in chitosan nanoparticles; pPCFN-CS-NPs, the pVAX1-PCFN DNA plasmid encapsulated in chitosan nanoparticles

To characterize the morphology and stability of pPCFN-CpG-CS-NPs, the chitosan-DNA nanoparticles were prepared by a complex coacervation method. Typical pPCFN-CpG-CS-NPs showed spherical and poly-disperse nature as revealed by TEM (Fig. 2a). The morphology of the pPCFN-CpG-CS-NPs had regular round shapes, smooth surfaces, and good dispersion, and did not have adhesion or subsidence damage. The average diameter was 93.58 nm and the size distribution by volume, number and intensity mean were 69.62 nm, 36.11 and 138.6 nm, respectively (Fig. 2b–d and Additional file 1: Table S1). The particle-size dispersity was 0.278 and the zeta potential was + 5.27 mV (Fig. 2e and Additional file 1: Table S1). The naked plasmid DNA was degraded within 30 min of incubation with DNase I (Lane 6 of Fig. 2f). The plasmid DNA encapsulated in chitosan nanoparticles was protected from degradation by DNase I and chitosanase (Lanes 1, 2, and 5 of Fig. 2f). The results demonstrated that chitosan encapsulation protected the DNA from DNase I digestion.

### Expression of pPCFN-CpG-CS-NPs and multivalent DNA plasmids in vitro

**Fig. 3:**
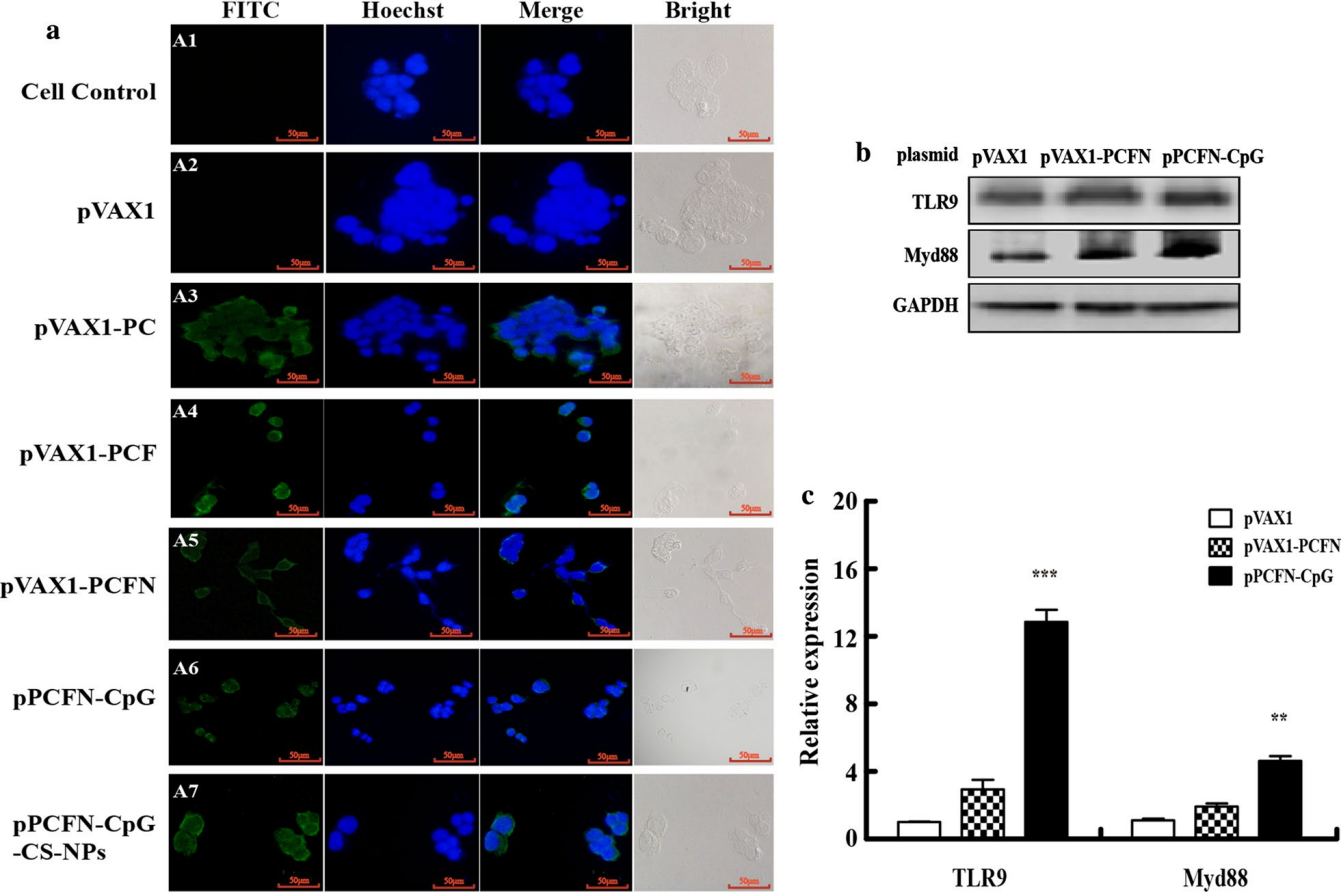
Transient expression of the chimeric protein and activation of CpG. **a** HEK293T cells were transfected with plasmid pPCFN-CpG, pVAX1-PCFN, pVAX1-PCF, pVAX1-PC, pVAX1, or pPCFN-CpG-CS-NPs respectively. Transient expression of proteins was detected with anti-PLO mouse polyclonal antibody by using fluorescence microscopy assays (A3-A7). (A1) Cell controls. (A2) Cells were transfected with vector pVAX1. FITC: FITC-conjugated goat anti-mouse IgG; Hoechst: cell nuclei; Merge: the overlay fluorescence images of FITC and nucleus; Bright: the external profiles of HEK293T cells. The scale bar is 50 μm. **b** and **c** RAW264.7 were transfected with pVAX1, pVAX1-PCFN or pPCFN-CpG plasmid for 36 h. The expression levels of TLR9 and Myd88 were detected by western blotting (**b**) and qPCR (**c**). Data are shown as the mean ± SEM of three independent experiments. ***p < 0.001 and **p < 0.01

To verify expression of the target protein in mammalian cells, IFA analysis of transfected HEK293T cells was performed. We found that the green fluorescence (Fig. 3a, A3–A7) was detected successfully by inverted fluorescence microscopy for HEK293T cells transfected with plasmids pPCFN-CpG, pVAX1-PCFN, pVAX1-PCF and pVAX1-PC or pPCFN-CpG-CS-NPs. In contrast, such effects were not observed in the cells transfected with empty vector pVAX1 and cell control (Fig. 3a, A1, A2). Additionally, we performed IFA to validate the expression of CbpA, FimA, and NanH by using rabbit anti-*T. pyogenes* polyclonal antibody according to previous reports [8, 35]. The results showed that the green fluorescence was also detected by inverted fluorescence microscopy for HEK293T cells transfected with plasmids pVAX1-PC, pVAX1-PCF, pVAX1-PCFN, and pPCFN-CpG (Additional file 2: Figure S1). It is well documented that TLR9 has evolved to recognize bacterial infections by the frequently unmethylated CpG DNA [22]. To investigate the activation of CpG, we measured the expression of TLR9 and Myd88 by using western blotting and qPCR. Our results showed that transient transfection of pPCFN-CpG increased the amount of TLR9 and Myd88 protein in RAW264.7 compared to pVAX1 or pVAX1-PCFN treated RAW264.7 (Fig. 3b). Similarly, qPCR data showed transfection with pPCFN-CpG significantly enhanced the mRNA expression of TLR9 and Myd88 in RAW264.7 compared to pVAX1 or pVAX1-PCFN treated RAW264.7 (Fig. 3c). Taken together, these data suggested that chimeric proteins were expressed in HEK293T cells and further validated the activation of CpG as an adjuvant.

### pPCFN-CpG-CS-NPs and multivalent DNA vaccines promoted antibody production

**Fig. 4:**
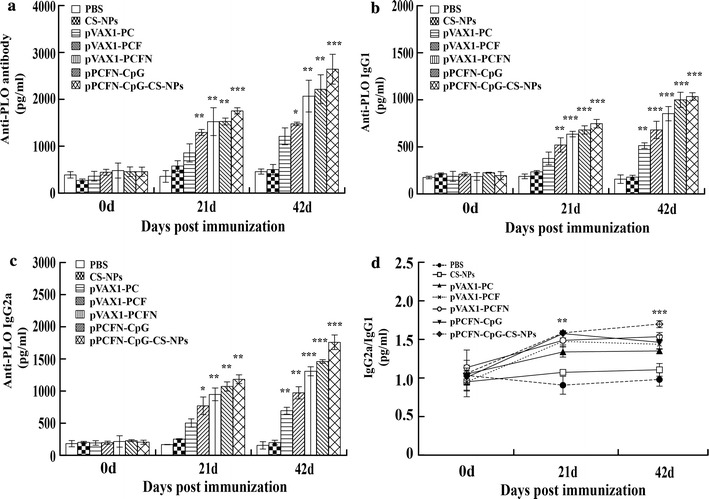
Humoral immune responses induced by the plasmids. Mice were immunized with plasmid pPCFN-CpG, pVAX1-PCFN, pVAX1-PCF, pVAX1-PC, or pPCFN-CpG-CS-NPs respectively, and boosted once with a 3-week interval. PBS was used as a control. Serum samples were collected from the tail vein of mice at the indicated time points. The titers of the PLO-specific antibody were detected by indirect ELISA assay. **a** Serum total IgG titers against PLO in immunized mice; **b** serum IgG1 titers against PLO in immunized mice; **c** serum IgG2a titers against PLO in immunized mice; **d** the dynamic changes of serum IgG2a/IgG1 against PLO in immunized mice. Data represented mean ± SD of OD at 490 nm. *p < 0.05 and **p < 0.01 (determined by one-way ANOVA followed by a Tukey–Kramer post hoc test)

To examine whether the immunogenicity of a multivalent chitosan-DNA nanoparticles vaccine targeting *T. pyogenes* was greater than that elicited by a bivalent DNA vaccine, we prepared four different plasmids and chitosan-DNA nanoparticles, each encompassing the *plo*, *cbpA*, *fimA*, or *nanH* gene. The mice were then immunized via intramuscular injection with either PBS and CS-NPs control, bivalent plasmid, or multivalent plasmids and pPCFN-CpG-CS-NPs. Antibody responses specific for the target antigen were examined by using ELISA. As shown in Fig. 4a, vaccination with the plasmid pPCFN-CpG, pVAX1-PCFN induced the production of PLO-specific antibody in sera at 21 dpi. When the mice were immunized with pPCFN-CpG-CS-NPs, the antibody could be detected since 21 dpi showed a higher titer on the indicated time points (*p* < 0.05), suggesting multivalent chitosan-DNA nanoparticles vaccine induced higher hormone immune response than bivalent DNA vaccines. No or low antibody titers in sera could be detected in CS-NPs or PBS groups. The serum antibody titers IgG1 and IgG2a subtype were monitored at regular intervals by ELISA. Likewise, the group of pPCFN-CpG-CS-NPs showed a higher level than other groups (*p* < 0.05) (Fig. 4b, c). As shown in Fig. 4d, serum antibody titers IgG2a was higher than IgG1 subtype, suggesting that these type of plasmids and pPCFN-CpG-CS-NPs were mainly priming Th1 type immune response.

### pPCFN-CpG-CS-NPs and multivalent DNA vaccines enhanced cellular immune response

**Fig. 5:**
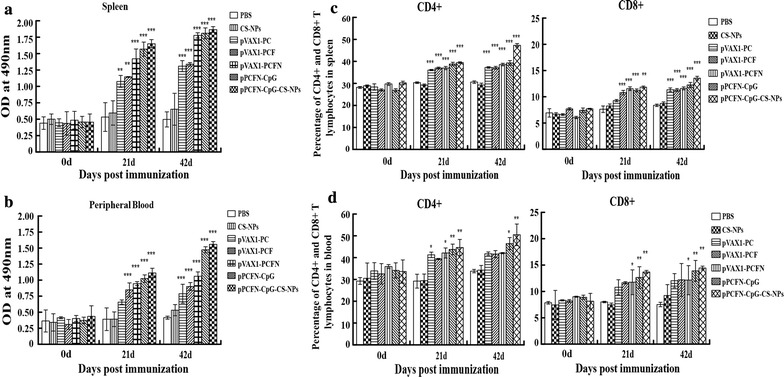
Proliferation of T lymphocytes and changes of CD4^+^ and CD8^+^ T lymphocytes in spleens and the peripheral blood. The T lymphocytes from the spleen (**a**) and peripheral blood (**b**) of mice were separated and their proliferation was determined by MTT assay. The CD4^+^ and CD8^+^ T Lymphocytes were separated from the spleen (**c**) and peripheral blood (**d**) at the indicated time points and were sorted by flow cytometry. PBS was used as a control. Data represented mean ± SD. *p < 0.05, **p < 0.01, and ***p < 0.001 (determined by one-way ANOVA followed by a Tukey–Kramer post hoc test)

The changes of CD4^+^ and CD8^+^ T lymphocyte and the levels of IFN-γ, IL-2, and IL-4 induced by the plasmids were investigated to evaluate the cellular immune response. The results showed that immunization with pPCFN-CpG-CS-NPs induced more potent lymphocyte proliferation and stimulated a higher level of IFN-γ, IL-2, and IL-4 in immunized mice than other groups (Fig. 5).

The results of MTT assay showed that the values from the group of pVAX1-PC, pVAX1-PCF, pVAX1-PCFN, and pPCFN-CpG or pPCFN-CpG-CS-NPs were increased between 21 and 42 dpi. However, such effects were not detected in the CS-NP or PBS groups (Fig. 5a, b). Immunization with pPCFN-CpG- CS-NPs induced more lymphocytes in spleen when compared with other groups (*p* < 0.001) (Fig. 5a). Likewise, the proliferation of lymphocytes in peripheral blood showed a similar tendency between 21 and 42 dpi (*p* < 0.001) (Fig. 5b).

Flow cytometry was performed to investigate the changes of CD4^+^ and CD8^+^ T lymphocytes in the spleen and blood. Compared to the control groups (Fig. 5c), the percentages of CD4^+^ and CD8^+^ T lymphocytes in spleens gradually increased after immunized with plasmids pVAX1-PC, pVAX1-PCF, pVAX1-PCFN, and pPCFN-CpG (*p* < 0.001) or immunization with pPCFN-CpG-CS-NPs (*p* < 0.001) between 21 dpi and 42 dpi. The group of pPCFN-CpG-CS-NPs showed a higher level than other groups (Fig. 5c). A similar result was also observed in the changes of CD4^+^ and CD8^+^ T lymphocytes in the blood between 21 dpi and 42 dpi (Fig. 5d).

**Fig. 6:**
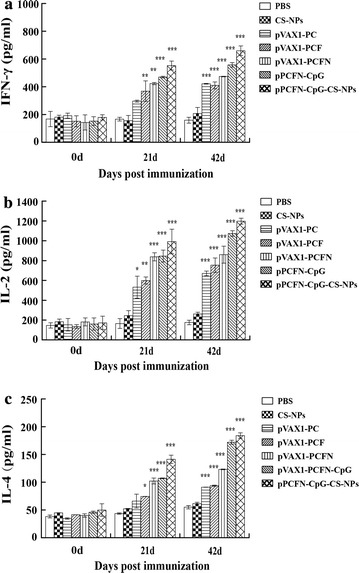
Levels of IFN-γ, IL-2, and IL-4 from the suspension of the spleen lymphocytes in immunized mice. The levels of IFN-γ (**a**), IL-2 (**b**), and IL-4 (**c**) from the suspension of the spleen lymphocytes were determined by ELISA assays. PBS was used as a control. Data represented mean ± SD. *p < 0.05, **p < 0.01, and ***p < 0.001 (determined by one-way ANOVA followed by a Tukey–Kramer post hoc test)

The levels of IFN-γ, IL-2, and IL-4 in the immunized mice were analyzed using ELISA. The results showed that immunization with pVAX1-PC, pVAX1-PCF or pVAX1-PCFN alone enhanced the levels of IFN-γ, IL-2, and IL-4 in the suspension of the spleen lymphocytes. In addition, the levels of IFN-γ, IL-2, and IL-4 were promoted with the vaccination of CpG motifs or encapsulated in chitosan nanoparticles (Fig. 6). Immunization with pPCFN-CpG-CS-NPs triggered the higher levels of IFN-γ, IL-2, and IL-4 between 21 dpi and 42 dpi in comparison to other groups (*p* < 0.001) (Fig. 6a–c). No significant changes of IFN-γ, IL-2 or IL-4 were observed in the suspension of the spleen lymphocytes of the mice immunized with CS-NPs or PBS (Fig. 6a–c). Taken together, these data clearly demonstrated that chitosan-DNA nanoparticles vaccine induced effective immune responses and the construction of the CpG ODN could function as a molecular adjuvant in conjunction with DNA immunogens to improve the cellular immune responses.

### pPCFN-CpG-CS-NPs and multivalent DNA vaccines protected mice from *T. pyogenes* challenge

**Fig. 7:**
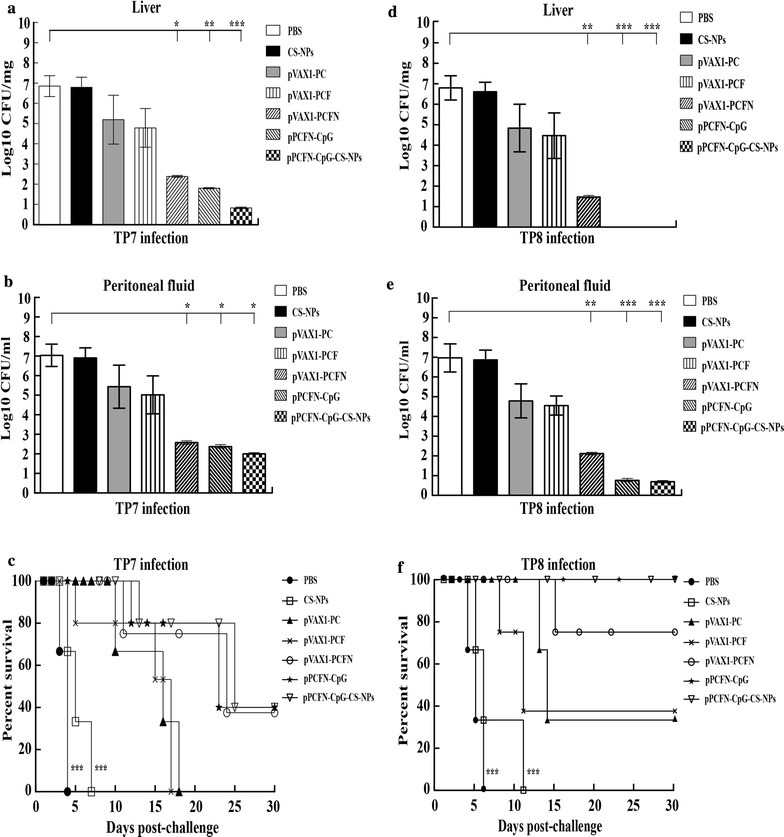
Residual CFUs and survival rate of mice challenged intraperitoneally with *T. pyogenes*. The mice were intraperitoneally challenged at day 43 with 3.7 × 10^8^ CFU *T. pyogenes* TP7 and *T. pyogenes* TP8 for determining liver (**a**, **d**) and PF (**b**, **e**) bacterial burdens. Survival rate was monitored for the subsequent 30 days (**c**, **f**). PBS was used as a control. *p < 0.05, **p < 0.01 and ***p < 0.001 (determined by Mantel–Cox log rank test)

**Fig. 8:**
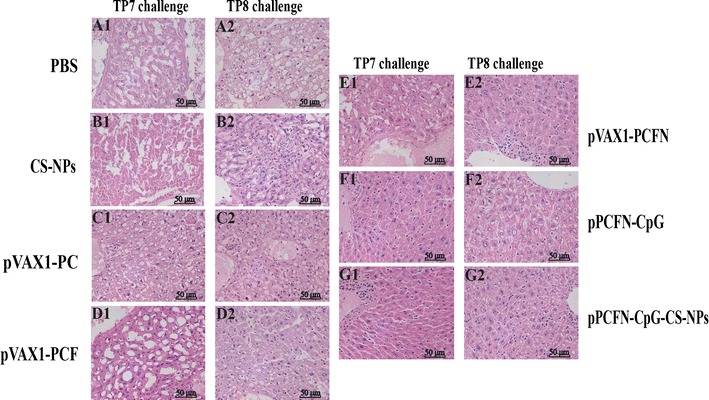
Histological analysis of mice challenged intraperitoneally with *T. pyogenes* TP7 and *T. pyogenes* TP8. The mice were intraperitoneally challenged at day 43 with 3.7 × 10^8^ CFU *T. pyogenes* TP7 (A1-G1) or *T. pyogenes* TP8 (A2–G2), respectively. The livers of seven groups of immunized mice were collected for evaluation of histology alteration. PBS was used as a control

To assess the ability of chitosan-DNA vaccine-raised immunity to eliminate *T. pyogenes* infection, the CFU of viable bacteria in the liver or PF was examined after intraperitoneal challenge with *T. pyogenes*. In the mice that challenged with 3.7 × 10^8^ CFU of *T. pyogenes* TP8 and *T. pyogenes* TP7, large amounts of bacteria from the liver and PF were recovered in the PBS and CS-NP treated mice. In contrast, all the mice immunized with pPCFN-CpG-CS-NPs displayed few or no bacteria in the liver (*p* < 0.001) (Fig. 7a, d) and PF (*p* < 0.05 or *p* < 0.001) (Fig. 7b, e) at 30 days post infection (dpi). Additionally, multivalent DNA vaccination resulted in a significant clearance of bacteria compared to the bivalent DNA vaccine group (Fig. 7a, b, d, e). As shown in Fig. 7c, f, PBS and CS-NPs of immunized mice died at day 4 or day 7. However, almost half of the mice immunized with pPCFN-CpG and pPCFN-CpG-CS-NPs were survived the *T. pyogenes* TP7 challenge at 30 dpi (*p* < 0.001) (Fig. 7c). Importantly, most of the mice immunized with pVAX1-PCFN and 100% of mice immunized with pPCFN-CpG and pPCFN-CpG-CS-NPs displayed no disease symptom at any time and survived against *T. pyogenes* TP8 challenge at 30 dpi (*p* < 0.001) (Fig. 7f). Histologically, the mice livers of control group showed a necrotic center containing leukocytes, hepatocytes and cellular debris (Fig. 8A1, B1, A2, B2). However, the immunized mice significantly eliminated invading bacteria in their livers during *T. pyogenes* challenge and the liver tissue had no obvious pathological changes compared to control group (Fig. 8E1–G1, E2–G2). Furthermore, multivalent DNA vaccination showed less pathological changes compared to the bivalent DNA vaccine group (Fig. 8C1, D1, C2, D2). Altogether, these results suggest that pPCFN-CpG-CS-NPs and multivalent DNA vaccines effectively protected mice from *T. pyogenes* challenge.

## Discussion

As an opportunistic bacterium, *T. pyogenes* is related to miscellaneous pyogenic infections in animals [1–4]. The complex pathogenicity of *T. pyogenes* and the diverse function of its virulence factors represent major obstacles to the development of effective universal vaccines. In the present study, we successfully prepared chitosan nanoparticles containing the chimeric gene DNA vaccine by using an ionic cross-linking method. Interestingly, the chitosan-DNA vaccines induced strong humoral and cellular immune responses and had protective effects against the challenge of *T. pyogenes* infections. It is suggested that chitosan-DNA vaccines targeting the different components of bacteria can act synergistically to provide a host with the expanded range of protective immunity against *T. pyogenes*.

It has been demonstrated that biodegradable polymers could be used as a delivery vector for veterinary antigen genes and realize sustained release [40]. In this study, we prepared the chimeric gene DNA vaccine encapsulated in high-quality chitosan nanoparticles (pPCFN-CpG-CS-NPs). Under the optimal conditions, the pPCFN-CpG-CS-NPs showed a smooth surface, good dispersity, and no adherence or collapse phenomenon. In addition, the pPCFN-CpG-CS-NPs protected the plasmid DNA from DNase I digestion, as shown by electrophoresis after the enzyme digestion. In accordance with our findings, a recent study also found that chitosan nanoparticles were an effective delivery vector for DNA vaccines and realize sustained release [41]. Furthermore, the in vitro HEK293T cells or RAW264.7 transfected with the pPCFN-CpG-CS-NPs demonstrated that the expression of specific antigens was successfully detected and further confirmed the activation of CpG. Taken together, these findings demonstrated that the nanoparticle production procedure was safe, and the bioactivity of the plasmid DNA remained after the production of the nanoparticles.

The PLO-specific antibody was the major detection index of humoral immune responses because the antibody plays an important role in bacterial clearance and recovery of health [39]. Analyses of IgG antibody responses from the plasmids pPCFN-CpG, pVAX1-PCFN, pVAX1-PCF, and pVAX1-PC, or pPCFN-CpG-CS-NPs, and blank CS-NPs indicated that intramuscularly immunization with multivalent DNA vaccines induced stronger antibody responses than the control group. Surprisingly, immunization with pPCFN-CpG-CS-NPs induced more specific antibody in immunized mice than other groups. Intramuscularly immunization is very effective in eliciting humoral and cellular immune responses. Previous studies have suggested that CpG ODN enhances the immunity of the vaccine and chitosan provides longer residence times by intramuscular infection [25, 44, 45], which allows the vaccine to better access the lymphoid tissue and results in increased IgG production. Hence, our study further confirmed the stimulation effect of humoral immune responses of CpG ODN and chitosan in mice, compared with the background signal stimulated by chitosan solution and PBS.

Our previous study had demonstrated that the Th1 type immune response was considered to be the major host response used to contain *T. pyogenes* infection [39]. When the proliferation of lymphocyte and the changes of CD4^+^ and CD8^+^ T lymphocytes were investigated, we found that pPCFN-CpG-CS-NPs or pPCFN-CpG induced more potent lymphocyte proliferation in immunized mice than pVAX1-PCFN alone. Meanwhile, pPCFN-CpG-CS-NPs markedly increased more synthesis and release of IFN-γ, IL-2, and IL-4 than other groups. These interactions determine the protective mechanisms of the host that are essential against *T. pyogenes* infections and developing particular antibacterial immunity. In accordance with our findings, a recent study pinpointed that chitosan promoted dendritic cell maturation by inducing IFNs and enhanced cellular immunity [32]. Additionally, the particle size of chitosan plays an important role in regulating immune response. In an interesting report, small-sized chitin or chitosan particles were found to activate alveolar macrophages to express cytokines such as IL-12, tumor necrosis factor-*α* (TNF*α*), and IL-18, leading to INF-*γ* production [46]. Our study showed that small-sized pPCFN-CpG-CS-NPs induced more potent lymphocyte proliferation and cytokine production in immunized mice than control group, suggesting that chitosan has complex and size-dependent effects on immune responses. Therefore, we believe that nanoform may function as a potent adjuvant in conjunction with DNA immunogens to enhance the immune responses, which may provide a protection from *T. pyogenes* challenges.

To further investigate the protective effects of the microencapsulation of DNA vaccines (pPCFN-CpG-CS-NPs) and the effect of CpG DNA as the adjuvant against the challenge of *T. pyogenes*, we selected livers and PF of immunized mice as targets for detection of bacterial burdens. Akin to previous studies [39, 47] large amounts of bacteria were recovered from the liver and PF in the control group of treated mice, indicating that *T. pyogenes* could invade the mouse peritoneal cavity and related organs. By comparison, the mice immunized with pPCFN-CpG-CS-NPs displayed few bacteria in the liver and PF, suggesting that bacteria were cleared from the host and raised the survival rates of those mice. Importantly, immunization with pPCFN-CpG-CS-NPs could significantly protect mice from highly virulent *T. pyogenes* TP7 infection. Consequently, we conclude that DNA vaccine pPCFN-CpG encapsulated in chitosan induced effective immune responses and the construction of the CpG ODN could function as a molecular adjuvant in conjunction with DNA immunogens to improve immune responses and protect against *T. pyogenes* infections.

## Conclusions

In this study, we successfully prepared pPCFN-CpG-CS-NPs that induced significantly higher mucosal and humoral immune responses and had protective effects against the challenge of *T. pyogenes* infections based on a mouse model. Moreover, the biodegradable polymer nanoparticles chitosan protected the plasmid DNA from degradation and promoted the expression of the encapsulated plasmid DNA. These findings demonstrate that the CS-NPs encapsulated plasmid DNA is a safe and efficient drug release carrier system with immense potential for medical application in the future. More comprehensive studies by using controlled and targeted release of nanoparticles encapsulated DNA vaccine testing in large ruminants may contribute to controlling *T. pyogenes* diseases.

## Additional files

**Additional file 1: Table S1.** Size distribution and Zeta potential of the pPCFN-CpG-CS-NPs.

**Additional file 2: Figure S1.** Transient expression of chimeric protein in HEK293T using fluorescence microscopy assays. Transient expression of proteins was detected with anti-*T. pyogenes* rabbit polyclonal antibody (B-E). (A) Cell controls. FITC: FITC-conjugated goat anti-rabbit IgG; Hoechst: cell nuclei; Merge: the overlay fluorescence images of FITC and nucleus. The scale bar is 50 μm.

## Abbreviations

PLO: haemolytic exotoxin pyolysin
CbpA: collagen-binding protein
NanH: neuraminidases
FimA: fimbriae
IFNs: interferons
SPF: specific-pathogen-free
HEK293T: human embryonic kidney cells
CpG ODN: CpG oligodeoxynucleotides
TEM: transmission electron microscopy
CS-NPs: chitosan nanoparticles solutions
IFA: immunofluorescence assays
FITC: fluorescein isothiocyanate
TLR9: Toll-like receptor 9
Myd88: myeloid differentiation primary response 88
ELISA: enzyme-linked immunosorbent assay
MTT: (3-(4,5-dimethylthiazol-2-yl)-2,5-diphenyltetrazolium bromide)
PF: peritoneal fluid
