# Supplementary material for: Chitosan-DNA nanoparticles enhanced the immunogenicity of multivalent DNA vaccination on mice against Trueperella pyogenes infection
Source: J Nanobiotechnology. 2018 Jan 29;16:8. doi: 10.1186/s12951-018-0337-2 (PMC5787914; doi:10.1186/s12951-018-0337-2)
Supplement: Supplementary file 1 — Additional file 1: Table S1. Size distribution and Zeta potential of the pPCFN-CpG-CS-NPs. [file 12951_2018_337_MOESM1_ESM.docx]

**Table S1** Size distribution and Zeta potential of the pPCFN-CpG-CS-NPs.

| **Temperature** | **Z-Average** | **Volume Mean** | **Number Mean** | **Intensity Mean** | **PDI** | **Zeta potential** |
| --- | --- | --- | --- | --- | --- | --- |
| **℃** | **d. nm** | **d. nm** | **d. nm** | **d. nm** |  | **mV** |
| 25.0 | 93.58 | 69.62 | 36.11 | 138.6 | 0.278 | 5.27 |
