# Supplementary figures and images for: Chitosan-DNA nanoparticles enhanced the immunogenicity of multivalent DNA vaccination on mice against Trueperella pyogenes infection
Source: J Nanobiotechnology. 2018 Jan 29;16:8. doi: 10.1186/s12951-018-0337-2 (PMC5787914; doi:10.1186/s12951-018-0337-2)

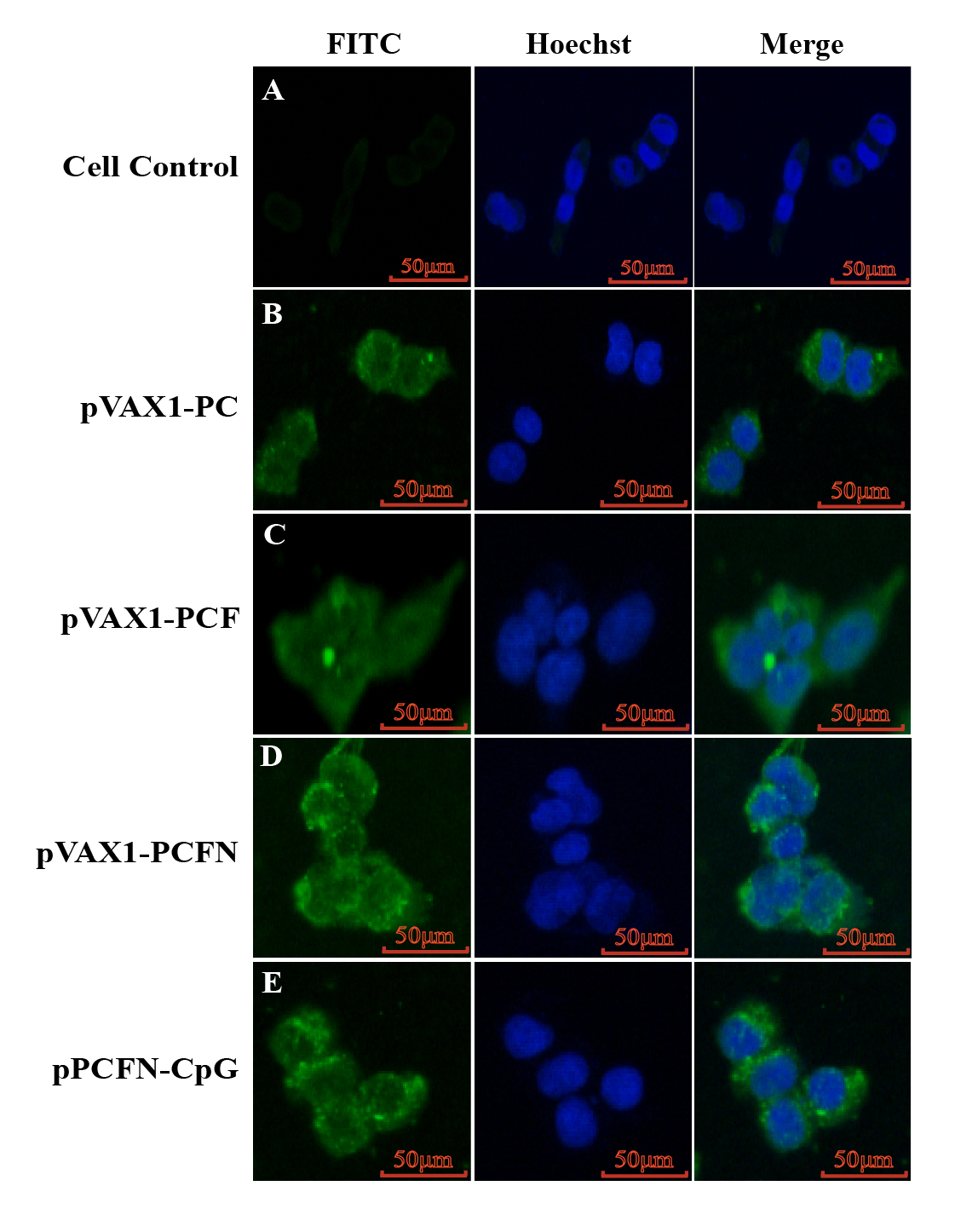

Supplement: Supplementary file 2 — Additional file 2: Figure S1. Transient expression of chimeric protein in HEK293T using fluorescence microscopy assays. Transient expression of proteins was detected with anti-T. pyogenes rabbit polyclonal antibody (B-E). (A) Cell controls. FITC: FITC-conjugated goat anti-rabbit IgG; Hoechst: cell nuclei; Merge: the overlay fluorescence images of FITC and nucleus. The scale bar is 50 μm. [file 12951_2018_337_MOESM2_ESM.tif]
